# The Chemical Composition and Transcriptome Analysis Reveal the Mechanism of Color Formation in Tea (*Camellia sinensis*) Pericarp

**DOI:** 10.3390/ijms241713198

**Published:** 2023-08-25

**Authors:** Yueyang Du, Yongen Lin, Kaikai Zhang, Dylan O’Neill Rothenberg, Huan Zhang, Hui Zhou, Hongfeng Su, Lingyun Zhang

**Affiliations:** College of Horticulture, South China Agricultural University, Guangzhou 510640, China; dyyscau@gmail.com (Y.D.); yongenlin@163.com (Y.L.); zhangkaikai1010@163.com (K.Z.); dylan.rothenberg@colorado.edu (D.O.R.); 20222018006@stu.scau.edu.cn (H.Z.); zh15879334458@163.com (H.Z.); 15219649384@163.com (H.S.)

**Keywords:** *Camellia sinensis*, pericarp, coloration, anthocyanin, albino

## Abstract

To elucidate the molecular mechanisms underlying the differential metabolism of albino (white), green, and purple pericarp coloration, biochemical profiling and transcriptome sequencing analyses were performed on three different tea pericarps, Zhongbaiyihao (*Camellia sinensis* L. var. Zhongbai), Jinxuan (*Camellia sinensis* L. var. Jinxuan), and Baitangziya (*Camellia sinensis* L. var. Baitang). Results of biochemical analysis revealed that low chlorophyll content and low chlorophyll/carotene ratio may be the biochemical basis for albino characteristics in the ‘Zhongbaiyihao’ pericarp. The differentially expressed genes (DEGs) involved in anthocyanin biosynthesis, including DFR, F3′5′H, CCoAOMT, and 4-coumaroyl-CoA, were highly expressed in the purple ‘Baitangziya’ pericarp. In the chlorophyll synthesis of white pericarp, GUN5 (Genome Uncoupled 5) and 8-vinyl-reductase both showed high expression levels compared to the green one, which indicated that albino ‘Zhongbaiyihao’ pericarp had a higher chlorophyll synthesis capacity than ‘Jinxuan’. Meanwhile, chlorophyllase (CLH, CSS0004684) was lower in ‘Baitang’ than in ‘Jinxuan’ and ‘Zhongbaiyihao’ pericarp. Among the differentially expressed transcription factors, MYB59, WRKY41-like2 (CS ng17509), bHLH62 like1 (CS ng6804), and bHLH62-like3 (CSS0039948) were downregulated in Jinxuan pericarp, suggesting that transcription factors played a role in regulating tea pericarp coloration. These findings provide a better understanding of the molecular mechanisms and theoretical basis for utilizing functional components of tea pericarp.

## 1. Introduction

Tea (*Camellia sinensis* (L.) O. Kuntze) is one of the most important economic crops in South China. In recent years, tea production has become a powerful driver of rural revitalization in less-developed mountainous regions in Southwest China. In efforts to enhance tea production, an enormous amount of research has been conducted on the primary metabolic processes involved in bud and leaf development in different tea varieties [1]. Fruits of tea plant, on the other hand, are often dismissed, with the exception of small amounts used in propagation and oil extraction [2]. Due to the lesser economic value of pericarp compared with young shoots, significantly less research has focused on secondary metabolism during pericarp development. Specifically, albino pericarp is a rare mutation in tea plants, which has not been reported yet.

The fruit of the tea is a capsule that can be divided into three parts based on resource utility: the pericarp, the seed husk, and the seed kernel [2]. Chlorophyll is an important pigment in the chloroplast involved in photosynthesis, and significant changes in its contents can lead to changes in plant color [3]. The albino phenotype is a leaf color mutation that has been identified in a variety of plants, including Arabidopsis [4], maize [5] and rice [6]. Normally, the biosynthesis and degradation of chlorophylls in plants are in dynamic equilibrium. Once the expression levels of genes involved in the chlorophyll degradation pathway change, this balance can be disrupted, leading to abnormal pericarp color [7]. Numerous studies have shown that carotenoids, flavonoids, and alkaloids play critical roles in the formation of plant color [8]. In particular, carotenoids and flavonoids have received particular attention due to their ability to produce bright colors on flower petals and leaves and because of their wide distribution across plants. Carotenoids provide flowers with a range of colors from orange to yellow [9], while flavonoids have been considered the most important secondary metabolites in plants [10]. Anthocyanins are important water-soluble compounds extensively distributed throughout the plant kingdom that provide fruits and flower tissue a red to blue color [11]. In research studies on ornamental plants of the genus Camellia or the family Camelliaceae, anthocyanin chemical composition and color have been widely studied [12]. Moreover, anthocyanins are among the main taste mediators of bitterness in tea [13]. Exploring the similarities between the coloring mechanism of pericarp and leaves of tea and investigating the accumulation mechanism of anthocyanin and other pigments in pericarp may be beneficial to improving the quality of tea production.

Transcriptome high-throughput sequencing (RNA-Seq) technology has been widely used to study the secondary metabolic mechanism of crops [14]. Analysis of gene expression profile by high-throughput sequencing technology is an effective method to study the overall gene expression difference from the transcriptional level. Its advantage is that it can analyze the transcriptional expression profile of new species without a genomics background or cDNA cloned by bacteria and effectively discover and identify target genes [15]. Transcriptomics has opened up new approaches for tea genetic research and can also be used to create markers to assist in breeding, for reducing the cost of marker selection and shortening the breeding process [16,17,18]. In recent years, anthocyanin-rich tea varieties, such as Zijuan (*Camellia sinensis* var. assamica) have been extensively studied for their anthocyanin accumulation mechanism in leaf tissue [19,20]; however, the coloration mechanisms of tea pericarp of different varieties remains unclear. Studies have suggested that albino mutants in plants may be formed by a variety of factors, including reductions in chlorophyll anabolism [10,21]. Reduced chlorophyll content subsequently inhibits the development of the chloroplast, causing leaf whitening or yellowing [22].

In the present study, the chemical compositions of albino (Zhongbaiyihao), green (Jinxuan), and purple (Baitang) pericarps were analyzed, and the differentially expressed genes (DEGs) related to pericarp color formation were identified by RNA-seq. The aim of this study was to evaluate key compounds responsible for the pericarp color formation of different tea varieties, and to draw connections between well-studied mechanisms of vegetative growth and the lesser-known mechanisms of reproductive growth in tea.

## 2. Results

### 2.1. Pigment Content in Different Tea Pericarps

**Figure 1:**
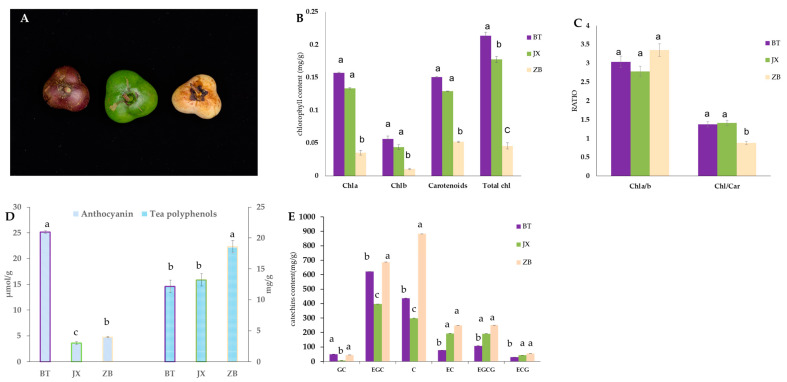
Phenotypic characteristics of Zhongbaiyihao (white), Jinxuan (green), and Baitangziya (purple) pericarp (**A**) and pigment contents of Zhongbaiyihao (ZB), Jinxuan (JX), and Baitangziya (BT) (**B**–**E**). Errors bar represent standard deviation of three independent replicates. Bars showing different lower-case letters indicate significant differences between groups (*p* < 0.05, one-way ANOVA, Student’s *t*-test). BT is ‘Baitang’ (purple pericarp), JX is ‘Jinxuan’ (green pericarp), ZB is ‘Zhongbaiyihao’ (white pericarp). C, catechin; EC, epicatechin; ECG, epicatechin gallate; GC, gallocatechin; EGC, epigallocatechin; GCG, gallocatechin gallate; ECGC, epigallocatechin gallate.

Phenotypic characteristics of different tea pericarp colors revealed that the pericarp of JX was a normal green color, while ZB was white and BT was purple (Figure 1A). Consistent with phenotypic characteristics, the analysis of tea pericarp pigment contents showed that the levels of total chlorophyll in BT were higher than that of JX. The carotenoid content in the pericarp of ZB was significantly lower than that of the normal green variety JX (Figure 1B,C). The concentrations of tea polyphenols in the ZB pericarp were higher than BT and JX (Figure 1D), while the concentrations of anthocyanin were higher in BT than in JX and ZB (Figure 1E). Catechin analysis revealed that total catechins were higher in white pericarp (ZB), while EGC, GC, and C showed relatively higher levels in BT than in ZJ. The content of C (catechin) in ZB was significantly higher than JX and BT. 

### 2.2. Transcriptomic Profiling of Different Tea Pericarps 

**Figure 2:**
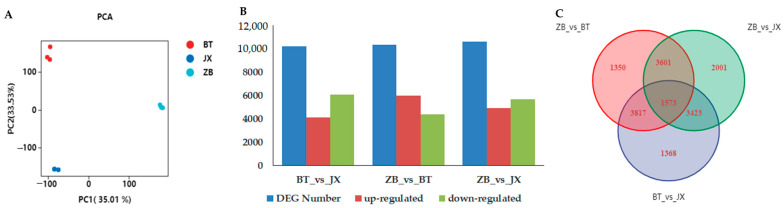
Differentially expressed genes (DEGs) in different tea pericarps. (**A**) Principle component analysis of pericarp samples indicates a high degree of clustering among intra-group samples. (**B**) DEGs among BT vs. JX, ZB vs. BT, ZB vs. JX. (**C**) Venn diagram of DEGs between three groups. BT is ‘Baitang’ (purple pericarp), JX is ‘Jinxuan’ (green pericarp), ZB is ‘Zhongbaiyihao’ (white pericarp).

To reveal the molecular mechanism of coloration in different pericarps, transcriptome analysis was performed using the different colored pericarps of ZB, JX, and BT. After data cleaning and raw read quality control, 60,293,150, 65,004,398 and 66,416,772 clean reads were obtained from the ZB, JX, and BT libraries, respectively, with clean data of 5.73 Gb for each sample, Q30 base percentages of 87.22% and above, and an average GC content of 44.73% (Supplementary Table S1). All clean data were mapped to the ‘Shuchazao2’ reference genome of *Camellia sinensis* var. sinensis (CSS, http://tpia.teaplants.cn/, accessed on 3 August 2022). Clean reads ranging from 72.90 to 89.49% were mapped (Supplementary Table S1). The PCA score plot of sequencing data is shown in Figure 2A and suggests that all pericarp samples were clustered and well separated into three groups. These results implied that the RNA sequencing data were reliable for subsequent analysis.

### 2.3. Identification of Differentially Expressed Genes between ZB, JX, and BT

To identify differentially expressed genes (DEGs) involved in color formation in different pericarps, fragmentation mapping in millions (FPKM) values for each gene in pericarps of ZB, JX, and BT were analyzed using a false discovery rate (FDR) ≤0.01 and fold change (FC) ≥2 as screening criteria. A total of 10,181, 10,341 and 10,598 DEGs were identified in pairwise comparisons of BT with JX, ZB with BT, and ZB with JX, respectively (Figure 2B). Both BT vs. JX and ZB vs. JX included more down-regulated DEGs than up-regulated genes. ZB vs. BT had significantly more up-regulated DEGs than down-regulated. The Venn analysis results showed that there were a total of 1573 common differentially expressed genes (FC ≥ 2, Q-value ≤ 0.05) among the three types of pericarp (Figure 2C). Among the common DEGs, HYC85, dehydrin DHN1-like, and oleosin 1-like showed the highest expression level in BT pericarp (Supplementary Table S2).

### 2.4. KEGG Enrichment Analysis

**Figure 3:**
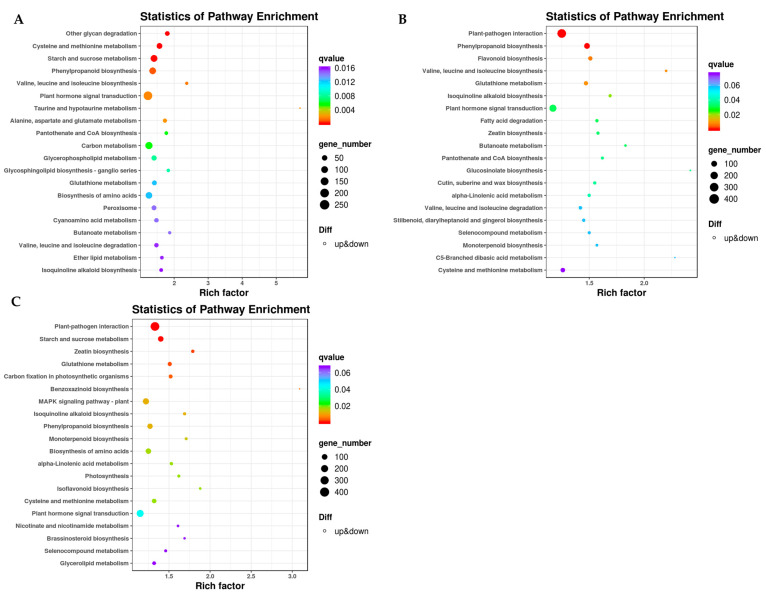
KEGG enrichment analysis of DEGs in different tea pericarp. JX vs. BT (**A**), ZB vs. BT (**B**), and ZB vs. JX (**C**). BT is ‘Baitang’ (purple pericarp), JX is ‘Jinxuan’ (green pericarp), ZB is ‘Zhongbaiyihao’ (white pericarp).

In order to determine the mechanisms of differential color formation in the three varieties of pericarp samples, DEGs involved in the flavonoid pathway, photosynthetic pathway, and the phenylpropane biosynthetic pathway were enriched and classified. In a pairwise comparison of BT and JX pericarp, DEGs in 117 biosynthetic and metabolic pathways were enriched, including phenylpropane biosynthesis (ko00940, 138 genes), flavonoid and flavonol biosynthesis (ko00944, 13 genes), flavonoid biosynthesis (ko00941, 56 genes), and anthocyanin biosynthesis (ko00942, 5 genes) (Figure 3A, Supplementary Table S3). In the paired comparison of ZB and BT pericarp DEGs, the genes were enriched to 117 KEGG pathways. DEGs involved in flavonoid biosynthesis included phenylalanine metabolism (ko00360, 31 genes), phenylpropanoid biosynthesis (k000940, 152 genes), flavonoid biosynthesis (ko00941, 69 genes), flavonol biosynthesis (ko0944, 15 genes), and anthocyanin biosynthesis (k00942, 3 genes) (Figure 3B, Supplementary Table S3). In comparison with JX, 119 DEGs in ZB were enriched in biosynthetic and metabolic pathways, including anthocyanin biosynthesis (ko00942, 6 genes), flavonoid biosynthesis (ko00941, 46 genes), phenylpropane biosynthesis (ko00940, 139 genes), photosynthesis (ko00195, 33 genes), and others (Figure 3C, Supplementary Table S3).

KEGG enrichment analysis indicated that 82 genes were up-regulated and 56 were down-regulated in the benzopropane biosynthetic pathway in BT and JX pairwise comparisons. A total of 38 genes were down-regulated in the flavonoid biosynthetic pathway in the ZB and BT pairwise comparison, and the remaining 45 genes were up-regulated. In the flavonoid and flavonol biosynthetic pathways, 15 DEGs were screened, 10 of which were up-regulated in white pericarps (ZB). In addition, three DEGs involved in the phenylpropane biosynthetic pathway were screened, namely, CCoAOMT (caffeoyl coenzyme A O-methyltransferase, CSS0043057), CCR (cinnamoyl coenzyme A reductase, CSS0026943), and the CsnewGene_15860 (Supplementary Table S3). The expression levels of these genes were significantly lower in white pericarp than in purple pericarp. 

In the photosynthetic pathway, photosystem II10 kDa polypeptide (PSII10 kDa polypeptide, CSS0001445), chlorophyll a-b binding protein 13 (CSS0015941), photosystem I subunit O (photosystem I subunit O (CSS0045008), photosystem I reaction center subunit psaK (CSS0016265), photosystem I reaction center subunit N (CSS0016265), photosystem I reaction center subunit N (CSS0010064), HY5 (HY5, CSS00048476) in the plant circadian rhythm pathway, and chlorophyllase (CLH, CSS00004684) in the porphyrin and chlorophyll metabolism pathway were also major DEGs. Excluding CLH, the expression levels of all the above DEGs in white pericarp (Zhongbaiyihao) were significantly higher than those in the conventional green pericarp (Jinxuan) (Supplementary Table S3). These data indicate a key role of DEGs in photosynthesis in photosystem I and photosystem II, as well as in response to light signals and regulation of participation in life activities on a circadian basis. 

### 2.5. Identification of DEGs Involved in Flavonoid Biosynthesis

**Figure 4:**
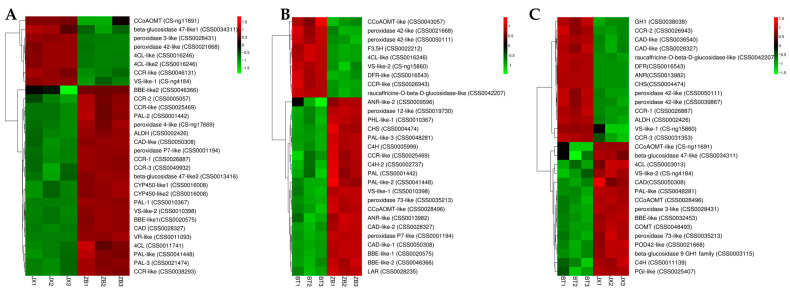
DEGs involved in flavonoid and phenylpropanoid biosynthesis in different tea pericarps. JX vs. ZB (**A**), ZB vs. BT (**B**), and BT vs. JX (**C**). BT is ‘Baitang’ (purple pericarp), JX is ‘Jinxuan’ (green pericarp), ZB is ‘Zhongbaiyihao’ (white pericarp).

In order to distinguish the critical genes involved in flavonoid and phenylpropanoid pathways in different pericarps, a clustering heat map was created to investigate the expression characteristics of relevant genes using KEGG enrichment analysis. Results showed that compared with ZB, the expression levels of CCoAOMT (CS ng11691), beta glucose 47-like 1 (CSS0034311), peroxidase3-like (CSS0028431), peroxidase 42-like (CSS0021668), 4CL-like (CSS0016246), 4CL-like 2 (CSS0016246), CCR-like (CSS0046131), and VS-like 1 (CS ng4184) were higher in JX pericarp (Figure 4A, Supplementary Table S4). Meanwhile, CCoAOMT like (CSS0043057), peroxidase 42-like (CSS0021668), peroxidase 42-like (CSS0050111), F3,5H (CSS0022212), 4CL-like (CSS0016246), VS-like 2 (CS-ng15860), DFR-like (CSS0016543), CCR-like (CSS0026943), and raucaffricine-O-beta-D-glucosidase-like (CSS0042207) showed higher expression levels in BT pericarp than in that of ZB (Figure 4B, Supplementary Table S4). Similarly, GH1 (CSS0038038), CCR-2 (CSS0026943), CAD like (CSS0036540), CAD like (CSS0028327), raucaffricine-O-beta-D-glucosidase like (CSS0042207), DFR (CSS0016543), ANR (CSS0013982), CHS (CSS0004474), peroxidase 42 like (CSS0050111), peroxidase 42 like (CSS0039867), CCR-1 (CSS0026887), ALDH (CSS0002426), VS-like 1 (S-ng15860), and CCR-3 (CSS0031353) were more highly expressed in BT than JX (Figure 4C, Supplementary Table S4). Comprehensive KEGG enrichment analysis showed that the expression level of genes involved in anthocyanidin synthesis in purple pericarp (BT) was high (such as DFR, ANR, etc.), while in white pericarp (ZB), the expression level of genes involved in downstream anthocyanidin synthesis pathway was relatively low.

### 2.6. Validation of Gene Expression Levels

**Figure 5:**
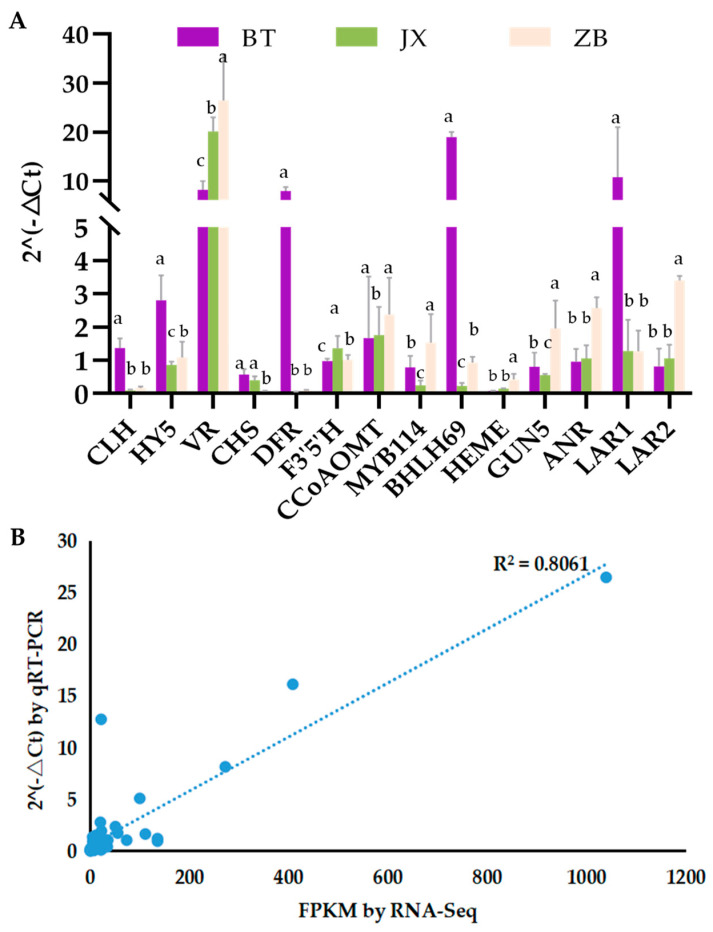
Expression analysis for key genes and transcription factors involved in pigment biosynthesis. (**A**) Expression level analysis of key genes and transcription factors in different pericarp. Errors bar represent standard deviation of three independent replicates. Bars showing different lower-case letters indicate significant differences between groups (*p* < 0.05, one-way ANOVA, Student’s *t*-test). (**B**) Correlation analysis based on RNA-seq and qRT-PCR data. CLH, Chlorophyllase; HY5, ELONGATED HYPOCOTYL5; VR, Vestitone reductase; CHS, Chalcone synthase; DFR, Dihydroflavonol reductase; F3′5′H, Flavonoid 3′,5′-hydroxylase; CoAOMT, Caffeoyl coenzyme A O-methyltransferase; HEME, Uroporphyrinogen III decarboxylase; GUN5, Genome Uncoupled 5; ANR, Anthocyanidin reductase; LAR, Leucoanthocyanidin reductase. BT is ‘Baitang’ (purple pericarp), JX is ‘Jinxuan’ (green pericarp), ZB is ‘Zhongbaiyihao’ (white pericarp).

Based on the RNA-Seq results of three different colored pericarps, eleven DEGs involved in color formation of tea pericarps were selected to confirm the accuracy of the RNA-Seq results using qRT-PCR (Figure 5A). Correlation analysis was performed to establish correlations between RNA-Seq and qRT-PCR results. The results revealed that the qRT-PCR and RNA-Seq data were highly correlated (correlation coefficient of 0.8171, Figure 5B). These results indicated that the transcriptome data were credible.

## 3. Discussion

### 3.1. Differences in Pigment Accumulation Lead to Differences in the Pericarp Color 

Previous studies revealed that leaf color formation in purple tea may be due to variations in flavonoid content [23,24]. While higher concentrations of chlorophyll are responsible for green leaf color and photosynthesis [3], albino or etiolated leaves are mainly attributed to lower chlorophyll and higher carotenoid concentrations in leaf tissue [10,21,25]. Some research suggested that carotenoids protect chlorophyll from photo-oxidative damage through certain reductive properties as well as the absorption and transfer of light energy in photosynthesis [26]. Therefore, if carotenoid synthesis is blocked, its chlorophyll protection capacity may be lost and eventually cause abnormal chloroplast development [27]. Some research has suggested that chloroplast in etiolated leaves is inhibited, thereby inhibiting the synthesis of chlorophyll [28]. Similar findings have been confirmed in specific photosensitive etiolated leaf cultivars ‘Yujinxang’ [21], ‘Huangjinya’ [29], and ‘Anjibaicha’ [30].

Similar results have been reported in studies on different fruit peels; the formation of the red color was proposed to be a combination of decreased chlorophyll and increased anthocyanin accumulation during litchi maturation [31,32], while the purple color of the mangosteen fruit pericarp was mainly due to anthocyanins [33]. In addition to anthocyanin, flavones and some flavonols also act as major pigments or co-pigments [31]. Flavonoids affect the colors of both fruits and vegetables as well as that of the grain pericarp [34,35,36]. In a study of sweet osmanthus pericarp, Han et al. found that lignans and phenolic acids were higher in green pericarps than in purple-black pericarps, while the opposite was true of anthocyanins and flavonoids [37].

The results of this study show that the total chlorophyll content of the white pericarp of ‘Zhongbaiyihao’ was significantly lower than the normal green pericarp of ‘Jinxuan’ and the purple pericarp of ‘Baitangziya’, with the Chl/Car ratio showing a similar trend (Figure 1C). Meanwhile, although the Chla/b of ZB was relatively high, the absolute content of chlorophyll in ZB was low (Figure 1B,C), which is not conducive to the formation of purple and green pericarps. Nearly all the chloroplast-related genes appeared to be highly expressed in the white pericarp, which may be related to the lower chlorophyll content in white pericarp and the need to synthesize related substances to maintain normal life activities. In addition, while the content of catechins is relatively high in ZB, catechins are coloress in plants, which could partially explain the white appearance of ZB pericarp. The carotenoid content varied in a similar way to the chlorophyll content of the three tea pericarp varieties. For BT with purple pericarp, high levels of anthocyanins, chlorophyll-a, chlorophyll-b, and carotenoids may be the main reasons for the formation of its purple phenotype (Figure 1B–D). Of course, because anthocyanins and catechins compete for substrate in the flavonoid synthesis pathway, the accumulation of anthocyanins in the purple pericarp reduced the substrate for catechin synthesis, resulting in less accumulation of catechins in the purple pericarp (Figure 1E). We also found that the expression of an R2R3-MYB, MYB114, was up-regulated in purple pericarp, suggesting that this gene may be involved in the regulation of anthocyanins during pericarp coloration (Supplementary Table S3). 

The present research results are consistent with previous studies – that is, the purple phenotype was positively correlated with total anthocyanin content [33,37,38,39]. Likewise, the low chlorophyll content in the white pericarp of ‘Zhongbaiyihao’ may be due to the abnormal synthesis and enhanced chlorophyll degradation. For Jinxuan, an adequate amount of chlorophyll may explain its green pericarp.

### 3.2. Expression of Different Structural Genes Affects the Synthesis of Chlorophylls, Carotenoids, and Flavonoids

Presently, the CCoAOMT, DFR, and F3’5’H genes are significantly up-regulated in the BT pericarp relative to Jinxuan, which is consistent with results of Liu et al. that found that the expression pattern of CCoAOMT was highly correlated with flavonoid concentrations in other plants [40,41]. Although CCoAOMT is not essential for anthocyanin accumulation, up-regulation of CCoAOMT may contribute to the production of more flavonoid derivatives in the pericarp. Flavonoid 3’,5’-hydroxylase (F3’5’H, CYP75A) is responsible for the conversion of the substrate dihydrokaempferol to dihydroquercetin [42], and dihydroflavonol reductase (DFR) is responsible for catalyzing the conversion of dihydromyricetin and dihydroquercetin to leucovorin [43]. Studies have shown that competition between the flavonoid/flavonol pathway and the anthocyanin pathway is primarily a substrate competition between the FLS and DFR, with FLS preferentially utilizing dihydroquercetin and dihydrokaempferolin [44]. FLS facilitates a metabolic shift towards flavonols, resulting in lower anthocyanin accumulation [12,45].

**Figure 6:**
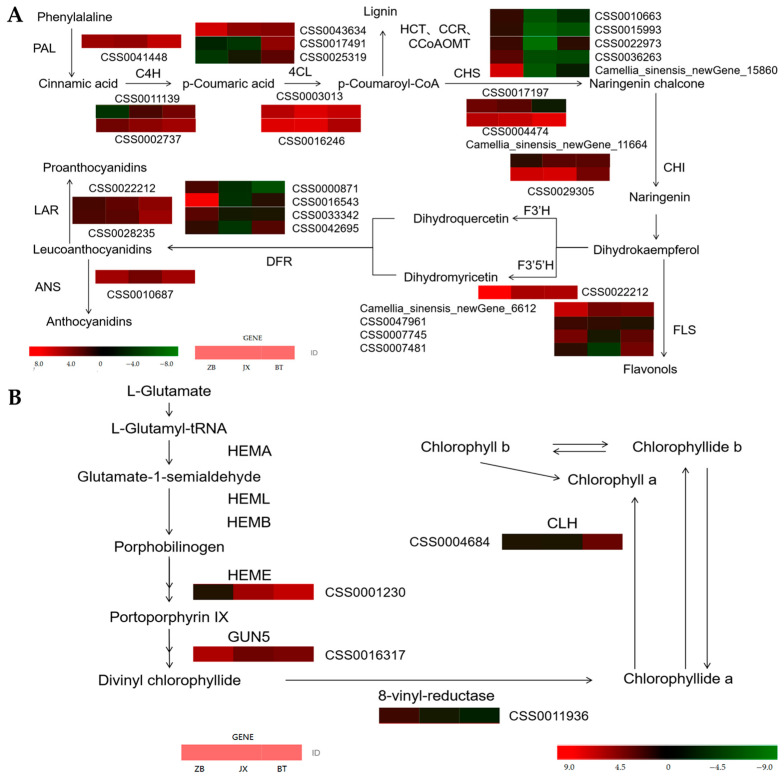
DEGs involved in the flavonoids and chlorophyll biosynthetic pathway. (**A**) DEGs involved in the flavonoids biosynthetic pathway; (**B**) DEGs involved in the chlorophyll biosynthetic pathway. Heat maps were created based on average expression levels (FPKM values). The color scale represents the FPKM value. Red indicates high expression, and green indicates low expression. BT is ‘Baitang’ (purple pericarp), JX is ‘Jinxuan’ (green pericarp), ZB is ‘Zhongbaiyihao’ (white pericarp). PAL, Phenylalanine ammonia-lyase; C4H, Cinnamate 4-hydroxylase; 4CL, 4-coumarate-CoAligase; HCT, Hydroxycinnamoyl-CoA shikimate/quinatehydroxy-cinnamoyltransferase; CCR, Cinnamoyl Co-A reductase; CoAOMT, Caffeoyl coenzyme A O-methyltransferase; CHS, Chalcone synthase; CHI, Chalcone isomerase; FLS, Flavonol synthase; F3’H, Flavonoid 3′-hydroxylase; F3’5’H, Flavonoid 3′5′-hydroxylase; DFR, Dihydroflavonol 4-reductase; LAR, Leucoanthocyanidin reductase; ANS, Anthocyanidin synthase; HEMA, Glutamyl-tRNA reductase; HEML, glutamate 1-semialdehyde aminotransferase; HEMB, porphobilinogen synthase; HEME, Uroporphyrinogen III decarboxylase; GUN5, Genome Uncoupled 5.

In this study, the higher expression levels of DFR indicates that flavonoid metabolism in purple pericarp shunted more substrate than green pericarp into the anthocyanin biosynthetic pathway (Figure 6A). The results of RNA-seq and qRT-PCR showed a relatively high FLS expression in green and white pericarp compared with the purple pericarp, implying that substrates might be shunted from the anthocyanins’ biosynthesis pathway towards the flavonol pathway in the substrate competition between FLS and DFR (Figure 6A).

Flavonoids and isoflavonoids are important secondary metabolites for plant defence that can function as inhibitors of fungal growth [46]. Vestitone reductase (VR) is a key reductase in the isoflavanone biosynthesis pathway. Previous studies found that VR activity corresponded with the synthesis of vestitone, which improved plant disease resistance [47,48]. The results of this study indicated that the VR gene has a high expression level, which may be due to unique isoflavanone metabolism in tea percarps. It is particularly noteworthy that ZB is a mutant variety of small leaf tea (Camellia sinensis var. sinensis) that was found previously to possess strong disease resistance, which may relate to its special accumulation of flavonoids. Moreover, we speculate that low anthocyanin and high tea polyphenol concentrations observed in ZB could relate to its high VR gene expression, because both anthocyanins and isoflavanones are metabolized through chalcone substrates. Such substrate competition could also affect the white appearance of ZB pericarp, because isoflavanones are colorless, unlike anthocyanins [49].

Meanwhile, we identified four DEGs related to the chlorophyll pathway, i.e., GUN5 (CSS0016317), HEME (CSS0012339), 8-vinyl-reductase (CSS0011936), and CLH (CSS0004684) (Figure 6B). Previous studies have reported that Genome Uncoupled 4 (GUN4) could bind ChlH/GUN5 to enhance chlorophyll biosynthesis by activating magnesium-chelatase, while the function of GUN5 is to shift protoporphyrin IX towards chlorophyll biosynthesis metabolism [50]. Meanwhile, HEME can catalyze uroporphyrinogen III to synthesize coproporphyrinogen III. Previous studies have shown that the content of heme and chlorophyll will decrease in transgenic tobacco with HEME-RNAi silenced [51]. Zhu et al. [52] confirmed that the levels of HEME expression in purple leaf tea were markedly different from green leaves, which resulted in variations in chlorophyll content. Similarly, our previous research also indicated that the lower expression levels of GUN5 and HEME resulted in the lower chlorophyll levels in yellow ‘Huangyu’ tea [53]. 

In this study, the chlorophyll concentration of white pericarp, in addition to higher GUN5 and 8-vinyl-reductase expression levels compared with green pericarp, indicated that ‘Zhongbaiyihao’ pericarp had a higher chlorophyll synthesis capacity than ‘Jinxuan’. Meanwhile, chlorophyllase (CLH, CSS0004684), which is involved in the chlorophyll degradation pathway, was higher in ‘Baitang’ than in ‘Jinxuan’ and ‘Zhongbaiyihao’ pericarps, suggesting that chlorophyll degradation activity in the pericarp of ‘Baitang’ was higher than those of ‘Jinxuan’ and ‘Zhongbaiyihao’ pericarps, resulting in reduced chlorophyll accumulation. The above results indicate that the higher chlorophyll level in ‘Zhongbaiyihao’ pericarp may be attributed to the higher expression of GUN5, HEME, and 8-vinyl-reductase (Figure 6B), while formation of the purple pericarp of ‘Baitang’ tea fruit may be due to the higher expression of DFR, which promotes the flow of substrates to the anthocyanin biosynthesis pathway.

### 3.3. Transcription Factors Involved in Pigment Accumulation

The content of anthocyanins is determined by structural genes as well as specific transcription factors (TFs) [54]. The structural genes of key enzymes in anthocyanin synthesis are subject to transcriptional regulation by transcription factors, and some of the more widely studied transcription factors include the MYB family, the bHLH family, and WD40 proteins, which regulate anthocyanin biosynthesis by binding to elements acting in the promoter regions of structural genes [55,56,57,58]. This has been well demonstrated in rice [59], cocoa [60], cotton [61], and several other plant species. 

In previous research, MYBs3 was found to be a single DNA-binding repeat MYB (R1-MYB) transcription factor that played a key role in cold adaptation in rice, likely by activating relevant genes when plants were subjected to various stressors [62]. Gan et al found that transgenic banana that over-expressed MpMYBS showed significantly higher cold tolerance than wild type, possibly by increasing proline levels in the transgenic banana line [63]. MYBs3 was also found to positively regulate anthocyanin biosynthesis during flower development in *Hibiscus syriacus* L. var. Shigyoku [64]. Wang et al. found that MYBs3 was involved in regulating resistance-related pathways in Eureka lemon, including phenylpropanoid, flavone/flavonol, and isoflavonoid pathways [65]. 

**Figure 7:**
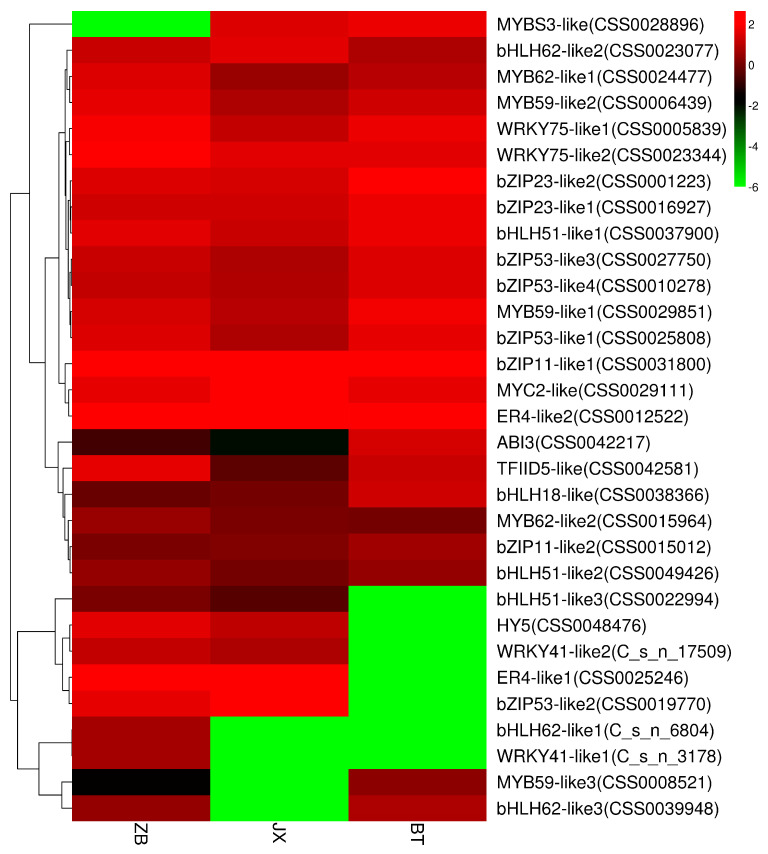
Differential expression of transcription factors involved in anthocyanidin and flavonoid pathways in different pericarps. The heatmap was created according to the expression levels of related transcription factors based on the FPKM value. The color scale represents the FPKM value. Red indicates high expression, and green indicates low expression. BT is ‘Baitang’ (purple pericarp), JX is ‘Jinxuan’ (green pericarp), ZB is ‘Zhongbaiyihao’ (white pericarp).

Presently, the expression of MYBS3-like (CSS0028896) transcription factor was significantly lower in ZB than in JX and BT (Figure 7). The reason may be that MYBS3-like was involved in regulating flavonoid biosynthetic processes in JX and BT pericarp.

Interestingly, transcription factors involved in suppressing anthocyanin biosynthesis were significantly downregulated in the ZJ pericarp, including bHLH51-like3 (CSS0022994), HY5 (CSS0048476), WRKY41-like2 (CS-ng17509), ERF4-like1 (CSS0025246), bZIP53-like2 (CSS0019770), bHLH62-like1 (CS-ng6804), and WRKY41-like1 (CS-ng3178) (Figure 7). In *Arabidopsis thaliana*, the WRKY41-1 transcription factor was significantly negatively correlated with anthocyanin levels, acting as a repressor of anthocyanin biosynthesis [66]. Similarly, MYB4-like (R2R3-MYB) and bHLH62 might also repress structural genes involved in anthocyanin synthesis, as it showed a significant negative regulatory relationship in the fruit skin of ‘Red Delicious’ [67]. The expression level of bZIP53 was up-regulated as anthocyanin levels decreased with fading flower color in the chrysanthemum, with a higher expression level of bZIP53 found to occur in response to high temperature [68]. Recent research found that ERF4 affected fruit firmness through TPL4 by reducing ethylene levels [69]. 

These findings discussed above indicate that the low expression of anthocyanidin transcriptional inhibitors may be related to the accumulation of anthocyanidin in BT pericarp. Correspondingly, due to the high expression of such transcriptional inhibitors, there was a reduction in the accumulation of flavonoids and anthocyanidins in white ZB pericarp. Among the transcription factors mentioned above, MYB59-like3 (CSS0008521), WRKY41-like2 (CS ng3178), bHLH62-like1 (CS ng6804), and bHLH62-like3 (CSS0039948) were down-regulated in JX purple pericarp. 

Lai et al. reported that the R2R3-MYB TF, MYB59, played a key role in some biological processes during development in Arabidopsis, negatively regulating leaf senescence when induced by jasmonic acid and salicylic acid in [70]. MYB59 can participate in circadian rhythm regulation by directly targeting CIRCADIAN CLOCK-ASSOCIATED 1 [71]. Other research showed that MYB59 could resist potassium deficiency stress by the positive regulation of Nitrate Transporter1.5 [72], represses calcium homeostasis, and regulates plant growth and stress response [73]. Wiśniewska et al. [74] reported that AtMYB59 may play an extensive role from metabolism modulation to the responses to abiotic and biotic stresses. Considering that the flavonoid content in the Jinxuan pericarp was in between ZB and ZJ, the down-regulation of the transcription factors mentioned above may be related to the metabolic balance of flavonoids, indicating that transcription factors play a role in regulating tea pericarp coloration.

### 3.4. Accumulation of Pericarp Pigments May Be Related to Light Induction

As a basic leucine zipper (bZIP) transcription factor, HY5 (ELONGATED HYPOCOTYL5) plays an important role in regulating plant growth and development by acquiring light signals through different light responsive cis-elements and transmitting them to downstream action elements [75]. It is the first transcription factor found to be involved in photomorphogenesis and plays a key role in regulating plant anthocyanidin biosynthesis. It was reported that HY5 might regulate GUN5 and HSP90 involved in chlorophyll biosynthesis [76]. Abbas et al. revealed that the HY5 transcription factor was involved in regulating nitrogen uptake and photogenesis, as well as assimilation in plants, and that the above regulatory mechanisms are completed through light response processes [77]. The function of the HY5 transcription factor is also related to phenotypes associated with photomorphogenesis in plants, such as hypocotyl elongation in seedlings and yellowing and de-yellowing of plants [78,79]. In addition, HY5 initiates downstream photomorphogenesis in photosensitive pigments, cryptochromes, and UV-B photoreceptors [80]. Furthermore, HY5 plays a critical role in chlorophyll accumulation and chloroplast development in plants, and can act as a central repressor in light signaling to enhance photomorphogenesis [81]. A recent study found that HY5 and CLH were up-regulated while *POR* and *HemA* genes were down-regulated in ‘Xiangfei Huangye’ etiolated tea, suggesting that chlorophyll synthesis was inhibited through increased expression of HY5, causing an increased rate of catabolism. [82]. 

In the present study, the expression profile of HY5 corroborated previous research results, in that the expression level of HY5 (CSS0048476) was higher in white pericarp than in the normal green pericarp. Interestingly, the expression level of HY5 was not significantly different between white ZB pericarp and purple ZJ pericarp. However, the expression level of CLH (CSS0004684) was significantly higher in the purple BT pericarp than the white and green pericarps. Considering that CLH can have a degrading function in the chlorophyll pathway, it appeared to show a negative effect on chlorophyll synthesis and accumulation. Therefore, in the purple pericarp, we speculate that the high expression level of CLH led to enhanced catabolism of chlorophyll, while the high levels of flavonoids promoted the shunting of upstream substances towards the anthocyanin metabolic pathway, resulting in the purple color of the pericarp. 

## 4. Materials and Methods

### 4.1. Plant Material

Tea (*Camellia sinensis* (L.) O. Kuntze) fruits were collected under natural conditions from tea plant varieties having white pericarp (*Camellia sinensis* var. Zhongbaiyihao), green pericarp (*Camellia sinensis* var. Jinxuan), and purple pericarp (*Camellia sinensis* var. Botangziya) grown in the tea garden of South China Agricultural University, Guangzhou, Guangdong Province, on 30 June 2022. The fruit samples were stripped of their pericarps and immediately frozen in liquid nitrogen, then promptly ground into powder and stored at −80 °C for further analysis. The samples were then subjected to further RNA-seq and chemical composition analysis. Each sample consisted of three biological replicates.

### 4.2. Extraction and Determination of Pigments

#### 4.2.1. Total Chlorophylls and Carotenoids

The total chlorophyll and carotenoids contents were determined by spectroscopy [83]. An amount of 500mg of sample was extracted in 25 mL of 95% ethanol (*v*/*v*) for 24 h at room temperature, and the extract was filtered and then fixed to 25 mL using 95% ethanol (*v*/*v*). The absorbance values were determined using an automatic microplate elisa reader (Sunrise, TECAN, Austria) as described by Rothenberg et al. [83]. The content of chlorophyll a, chlorophyll b, and carotenoids was calculated as follows:

Chl a = (12.21 × A663 − 2.81 × A646)/(1000 × W) × L;

Chl b = (20.13 × A646 − 5.03 × A663)/(1000 × W) × L;

Caro = (1000 × A470 − 3.27 × Chl a – 104 × Chl b)/(229 × 1000 × W) × L.

where A646, A663, and A470 indicate absorbance values at 646 nm, 663 nm, and 470 nm respectively; L indicates the total volume of the extract solution (mL), and W indicates the fresh weight (FW) of the sample (g); and the chlorophyll and carotenoid contents were determined in mg/g (FW).

#### 4.2.2. Determination of Total Anthocyanins

The total anthocyanin was determined as reported by Wei et al. [84]. An amount of 100 mg of sample powder was extracted in 3 mL of extraction solution (1% HCl in methanol) for 16 h at 4 ℃. The extract was filtered and then fixed to 10mL. The absorbance was measured at 530 nm and 657 nm by a spectrophotometer, using 1 cm cells. Total anthocyanin contents were calculated by the following formula: total anthocyanin = ((A530 − A620) − 0.1(A650 − A620) × 100)/(4.62 × W).

#### 4.2.3. Determination of Total Contents of Polyphenols

Total tea polyphenol contents were determined by the Folin reagent method, which is a modification of the method previously reported by Yu et al. [85]. Briefly, 200 mg of sample powder was extracted in 5 mL of extraction solution (70% methanol solution) in a 70 °C water bath for 10 min. Then, 1 mL extraction was supplemented with 5 mL Folin reagent to react for 8 min. Then, 4 mL of 7.5% Na_2_CO_3_ solution was added, and the reaction system was shaken for 60 min at room temperature. The absorbance of the reaction solution was measured at 765 nm by a spectrophotometer (UV-160 Shimadzu, Tokyo, Japan). The tea polyphenol concentration was quantitatively calculated with gallic acid as a calibration standard.

#### 4.2.4. HPLC Analysis of Catechins

The content of catechins was analyzed as described by Mei et al. [86]. Briefly, 200 mg of tea powders were extracted with 8 mL of 70% methanol in a 75 °C water bath for 30 min. The extraction solution was filtered through a 0.22 mm Millipore filter before conducting HPLC analysis. The chromatographic column was a C18 SB column (4.6 × 250 mm, 5 mm, Waters Technologies, Milford, MA, USA). The chromatographic conditions were as follows: Mobile phase A was decreased linearly from 92% in 5 min to 75% at 14 min and then increased linearly from 75% at 14 min to 92% at 30 min. Mobile phase A contained 0.1% acetic acid and 99.9% ultrapure water, and mobile phase B was 100% acetonitrile. The flow rate was 0.80 mL/min, and the column temperature was 30 °C; the injection volume was 20 µL. 

#### 4.2.5. RNA Extraction and Transcriptome Sequencing

Trizol (Invitrogen, Carlsbad, CA, USA) was used to extract total RNA from different pericarps [87]. The construction of a cDNA library and sequencing employed a Novaseq6000 by Biomarker Technologies Corporation (Beijing, China). The raw data were uploaded to Beijing Institute of Genomics, National Genomics Data Center, China (https://bigd.big.ac.cn/gsa, accessed on 3 September 2022), and the accession number is CRA007882. The raw data from the sequencing machines were initially filtered to obtain clean data by using SeqPrep software (Version 4.6.1). The HISAT2 program [88] was used to compare the clean reads of the filtered rRNA to the reference genome sequence (*Camellia sinensis* var. sinensis (CSS), ShuchazaoV2 genome, http://tpia.teaplants.cn/download.html, accessed on 3 August 2022).

### 4.3. Gene Function Annotation and Expression Level Analysis

In order to annotate the assembled genes, seven public databases were searched for homology, namely, NR (Non-Redundant Protein Sequence Database), KEGG (Kyoto Encyclopedia of Genes and Genomes), COG (Clusters of Orthologous Group of proteins), Swish-Prot database, KOG (Eukaryotic orthologous groups) database, Pfam (Pfam protein family database), and GO (gene ontology) database. The gene expression level was estimated by FPKM (every thousand base segments in the transcript mapped by every million segments). Based on the value of genes in the three varieties of samples, the differentially expressed genes were screened using DESeq2 software (version 1.38.0), with|log2FC| ≥ 1 and p-adj < 0.05 as the threshold. KEGG enrichment was performed using R-Package.

### 4.4. Quantitative Real-Time PCR and Expression Verification

In order to verify the RNA-Seq results, β- Actin was selected as an internal reference gene. The DEGs involved in chlorophyll metabolism and anthocyanin metabolism pathways were identified through transcriptome data analysis and the KEGG database, and eleven genes were screened for quantitative real-time PCR (qRT-PCR) verification. Total RNA was extracted using RNA Simple Total RNA Kit (TIANGEN) according to the protocol. The thermal profile for the PCR amplification was 95 °C for 5 min, and then 40 cycles of 10 s at 95 °C and 40 cycles of 30 s at 60 °C. The qRT-PCR primers are listed in Supplementary Table S5. Relative expression levels of the genes were quantified using the 2 ^−∆∆CT^ method [89].

### 4.5. Statistical Analysis

Statistical analysis was performed using SPSS software (version 24.0 for Windows, SPSS Inc., Chicago, IL, USA). Significant differences between different groups were determined using Tukey’s post hoc test. A *p* value less than 0.05 was considered statistically significant. Excel 2010 (Microsoft, Redmond, WA, USA) was applied to drawbar graphs of the experimental data. Some figures and tables related to transcriptomes were prepared on the BMKcloud platform (https://international.biocloud.net/, accessed on 29 May 2023).

## 5. Conclusions

In this study, chemical components and RNA-seq data were analyzed to explore the mechanisms underlying tea pericarp coloration. A total of 17,133 DEGs were identified. Some DEGs involved in the anthocyanin biosynthesis pathway, such as DFR, F3’5’H, CCoAOMT, and 4-coumaroyl-CoA, were highly expressed in purple BT pericarps, where they were positively correlated with the anthocyanin accumulation. In addition to the CLH gene, 33 DEGs involved in chlorophyll synthesis and degradation and 64 DEGs involved in photosynthesis-related proteins were identified. The low chlorophyll content of ZB may be due to the low expression level of HEME (heme A synthetase, CSS0016239) involved in chlorophyll synthesis and the high expression level of CLH involved in chlorophyll degradation. The high expression of CLH genes was negatively correlated with chlorophyll content in ZB pericarps. Multiple genes involved in repressing anthocyanin biosynthesis pathways were significantly down-regulated in the purple pericarp, including bHLH51-like3 (CSS0022994), HY5 (CSS0048476), WRKY41-like2 (CS-ng17509), and ERF4-like1 (CSS0025246). The expression level of HY5 (CSS0048476) was higher in the white pericarp than in the normal green pericarp, suggesting that transcription factors play a role in regulating the coloration of tea pericarp. Overall, the different colors in the pericarps of different tea varieties might be attributed to flavonoid and chlorophyll biosynthetic pathways. Our results provide new insights for clarifying the molecular mechanisms underlying pericarp coloration.

## Data Availability

The raw sequence data reported in this paper have been deposited in the Genome Sequence Archive in National Genomics Data Center, China National Center for Bioinformation/Beijing Institute of Genomics, Chinese Academy of Sciences (GSA: CRA007882) that is publicly accessible at https://ngdc.cncb.ac.cn/gsa, accessed on 21 September 2022.

## Supplementary Materials

The following supporting information can be downloaded at https://www.mdpi.com/article/10.3390/ijms241713198/s1.
